# Microchimerism and the need to rethink sex and gender binaries

**DOI:** 10.1186/s13293-025-00782-9

**Published:** 2025-11-17

**Authors:** Margrit Shildrick

**Affiliations:** https://ror.org/05f0yaq80 grid.10548.38 0000 0004 1936 9377 ERG, Stockholm University, SE-106 91 Stockholm, Sweden

The binary divisions that underpin the western logos are nowhere more evident than in the conventional beliefs that male and female, masculine and feminine, and indeed sex and gender themselves are all oppositions that demonstrate a clear distinction between one category and the other. With regard to the latter another binary split emerges insofar as sex is assuming to fall within the remit of the biological sciences while gender is a matter of social and cultural analysis. Such divisions may remain conveniences that simplify research strategies but in perpetuating normativities they do great damage to a deeper understanding of what mammalian life consists in. Feminist theory, emanating from both the biosciences and the humanities, has for some decades challenged the status quo and along that path has come to embrace a multidisciplinary approach that privileges neither nature nor nurture. At the same time developments in the field of biology have opened up a plethora of new ways of understanding the imbrication of sex and gender differences. The potential of such new approaches is nowhere more evident than in reassessing such issues as intersex and transgender forms of embodiment. As a biophilosopher, I focus here on what I see as the highly relevant phenomenon of microchimerism which grounds a largely speculative look towards a future in which the concepts of sex and gender are radically rethought.

Before explaining microchimerism, I want to swiftly review preceding analyses of the relationship between sex and gender, and more particularly how the morphological and psychological differences exemplified by intersex and transgender people have been understood. In its most uncritical version sex is deemed a matter of biology alone while gender is a social and cultural construction acquired through influences in early childhood. Although a spectrum of variations has long been accepted - see Fausto-Sterling [10, 11], Ainsworth [1] - the dominant attribution of sex itself as determined by the genetic input of both parents remains heavily biassed towards the binary exclusions of male and female. For the majority, the sex of a neonate appears to sit unproblematically at one end of the spectrum or the other, and where anomalies such as intersexed anatomy or a mismatch between chromosomal sex and anatomy occur, they are classified as disorders of sexual development [12].^1^ The further elaboration of masculinity and femininity supposedly follows from that initial sex binary such that an individual’s ‘failure’ to develop the anticipated gendered attributes warrants the label of transgender. Anne Fausto-Sterling’s now seminal work in promoting the model of the five-sex system where ‘chromosomes, hormones, the internal sex structures, the gonads and the external genitalia’ (2000: 20) are all highly variable is too modest a proposal and she and others have moved on to a realisation that no sex or gender variation is necessarily abnormal.

The notion that gender is a construction rather than something naturally given has been widely supported by feminist philosophers [4, 14, 38] who question the historically authoritative discourses of the biosciences, as do Heinämaa [18] and Richardson [37] who engage more closely with biology. All agree that nothing about embodiment is determined and Butler’s subsequent book – *Bodies that Matter* [5] – more pointedly expounds her critique of the sex/gender distinction itself. It is not just that gender is shaped by social, cultural, and political forces, but that material bodies themselves are subject to the same influences. The naming of biological sex at birth (or prenatally) is to a degree arbitrary, allowing little place for variations along the spectrum of potentially indivisible sex/gender differences. Increasing numbers of differently embodied individuals prefer to see themselves as fluidly transgender, swopping between masculine and feminine or refusing any sex or gender identity at all.

Given that sex and gender binaries have been shaken in recent years but by no means dismantled, I turn now to a development in the biological sciences that deeply disturbs the conventional understanding of how genetic coding works in human reproduction. The phenomenon of microchimerism is still seen as a relatively new discovery, though its operation in the human body has been known at least since the 1950 s and earlier in other mammals, including documentation of its effect in ruminants, especially with regard to freemartin cattle [35]. The term itself derives from the Greek myth of the chimera, a fabulous conjunction of a lion, a serpent and a goat that displays phenotypical attributes of each contributing creature. Microchimerism in contrast operates at the cellular level and refers to the occurrence of 2 or more different sets of DNA present in the same organism [25]. In human beings the term denotes a small but significant incidence of so-called non-self human cells coexisting with a dominant population of self cells in a singular body. More extensive *macro*chimerism may occur when the existing cells of the host body are outnumbered or even replaced, within a solid organ for example [39]. What microchimerism shares with its classical antecedent is the observation that there is no merging of differences of either distinct body parts in the case of the chimera or of differentially coded cells internal to the human body, but rather an enduring co-existence [40]. This does not imply that all non-self cells are functionally activated – except in clear cases of macrochimerism which may be located in specific sites such as the gonads – but they do introduce potential variation if expressed. The point is to propose uncertainty for philosophers and bioscientists alike. The science around microchimerism is still underdeveloped and projected causation is multiple but given that the area of greatest interest and sustained research lies in field of maternal-fetal relations, its implications for the attribution of a singular sex, male or female, are potentially game-changing.

What is perhaps surprising is that despite the recognition of (micro)chimerism in the plant kingdom as well as in farmyard cattle, acceptance of its existence in the human body has been slow and grudging. As far back as the mid-20^th^ century, Ray Owen [34] identified the most likely cause of the phenomenon of intersexual freemartin cows as the uterine exchange of chromosomal sex cells between male and female dizygotic twins, although the term chimerism was not used until a few years later, initially by the biologist Peter Medawar. The latter’s work on immunology – for which he subsequently shared a Nobel Prize – was instrumental in establishing the theory of the self/nonself cellular distinction and the supposed antagonism between the two. Although Medawar [30] recognised *natural* immuno-tolerance in some animals, the co-existence of microchimeric cells was at such odds with his immumological theory that he dismissed any occurrence as a ‘natural accident’ and ‘astonishing’. The first reported case of human microchimerism concerned a healthy blood donor, Mrs. McK, who was found to have 2 separate blood types, O and A, with the ‘alien’ type assumed to have derived in utero from the woman’s male twin [8]. Nonetheless, the dominance of Medawar’s immunological theory ensured that little progress was made in questioning how chimerism complicated the accepted model of self/nonself intolerance. Only in recent decades has the work of Thomas Starzl et al [42] on transplant recipients and seminal maternal-fetal research in labs headed by Diana Bianchi and Lee Nelson established the possibility of long-term chimerism. Along with biophilosopher Thomas Pradeu [36], they have demonstrated that a singular human body can accommodate genetically disparate cells as a matter of course [3, 47].

Bioscientific explanations of microchimerism are constantly evolving and while pregnancy may be the most frequent source of Y-coded cells in female-identified bodies, there are many known and possible alternative sources. All types of tissue, organ and bone marrow transplants, non-irradiated blood transfusions, generational genetic transfer and human dizygotic fusion^2^ are all likely candidates; and although untested, it has also been suggested by both clinical research and the popular media that lactation [31, 33, 50] and, more contentiously, fluid sexual exchanges can also generate microchimerism [7, 47]. Many such explanations are under-researched or speculative piecing together the implications of more established work, usually with an enduring focus on the immune system, yet the conventional notion of genetic inviolability becomes increasingly difficult to maintain. If each person in the normal course of life carries plural and durable populations of differentially active DNA, then the challenge to the model of genomically distinct bodies – and that must include the supposition that each organism is either male of female – is unavoidable. The hitherto unquestioned belief that human DNA is unvarying, persists over successive generations and defines each individual is no longer reliable. Chimeras disrupt the expectation of genetic uniformity and problematise the separation of self/non-self, and contest the binaries of male/female and masculine/feminine, such that the very notion of the bounded self falls away. Once the concept of genomic multiplicity is established the genetic basis for physical and cognitive development, health, disease, and identity are all challenged, and it becomes difficult to predict the limits of human plasticity or maintain trust in the inviability of any category of organism.

That the occurrence of microchimerism within a supposedly genetically unique body presents a serious challenge to the definitive principle of the immune system might seem self–evident. Nonetheless in the context of a socio-cultural imaginary that insists on clear boundaries between self and other, clinical discourse remains largely unchanged. The importance of reasserting immunity and assuring us of our continuing essential singularity largely shuts out recognition or interest in the wider implications. The seminal paper by Alexander et al [2], for example, carefully sets out evidence of extensive microchimerism, or more properly *macro*chimerism, in the case of a young female liver recipient who strongly coded for the DNA and Human Leucoyte Antigen (HLA) of the male donor,^3^ but it makes no comment on the resulting ambiguity in sexed/genetic identity. For biophilosophers, the evident hybridism of solid organs and the peripheral blood supply raises ontological issues that position cellular microchimerism as the starting point for a series of reconfigurations precisely about the genetic determination of sex. Whereas convention in biosciences may see the goal as finding a functional explanation as to why and how microchimerism occurs and whether its effects are beneficial or harmful, ephemeral or enduring, biophilosophical speculation on the problematic moves away from the binary divisions of singularity to open up to the concept of dynamic interconnections as a more convincing model for organic life, including human life. Transplantation raises many issues around microchimerism, but it is with the familiar processes of human reproduction that questions of sex and gender are most at stake.

It might be expected that the discovery in the late 1950 s of HLA would constitute a radical uncertainty about the projected success of any normal pregnancy. The understanding of HLA was quickly linked to the operation of the immune system [44] giving rise to the puzzle of why the differential HLA in place in maternal-fetal bodies does not lead, ordinarily, to mutual rejection. Nonetheless, that fundamental question surrounding the natural event of pregnancy has done little to shift the oppositional self/other paradigm [40, 41]. Unsurprisingly, the biomedical imaginary still characterises the immune system - as several feminist theorists have outlined [16, 29, 46], - in terms of militaristic metaphors expressing invasion, outright warfare and foreignness on the part of any incoming cellular agent being met by the resistance of a plethora of self-defence mechanisms. Nonetheless, the binary simplicity of that self/non-self distinction is potently challenged by the apparently *natural* tolerance between a pregnant woman and her fetus. As Martin summarises: ‘the imagery of aggressive immuno-warfare against the foreign focuses on the body that is all of one kind, all purely self…hence the normal woman would destroy her fetus to return to a normal state of internal purity’ [29]. The so-called ‘riddle of the fetal allograft’ has resulted in many explanations centring on specific mechanisms of immuno-tolerance peculiar to pregnancy which reinforce the view that the maternal-fetal bodies are still at base separate entities, immunologically opposed to another rather than enmeshed. The more recent understanding of microchimerism moves beyond the trope of invasion to focus on the complex and dynamic *interactions* between the maternal and fetal immune systems and provide a deeper insight into how pregnancy progresses to term despite the potential for immuno-rejection. As a radically different approach that does not rely on binary thinking, the microchimeric model has further implications in the matter of sex and gender.

Conventionally, the placenta has been recognised as a limited site of exchange between mother and fetus – oxygen, nutrients and hormones passing in one direction, products of excretion in the other – but is mainly seen as a protective barrier separating the distinct maternal and fetal bodies. In recent decades, however, a great deal of research – initially in Diana Bianchi’s lab – has uncovered strong evidence, now widely accepted, that both maternal and fetal cells cross the placental barrier as a matter of course effecting a kind of microchimerism within each body.^4^ The research is conducted, for convenience, by tracing the incidence of – conventionally out of place - Y-coded (‘male’) cells within the otherwise XX ‘female’ cellular body, and then generalised to other forms of maternal-fetal chimerism. Whilst few bioscientists now doubt the existence of microchimerism – ie small proportions of mismatched DNA in the host body - many prefer to see it as always transient and insignificant. Nonetheless, there is growing evidence that the process is not only routine, perhaps inevitable, in pregnancy, but persists throughout life in both mother and child [26]. Yet more significantly, Bianchi’s research detected that women who had never been pregnant could also be carrying the tell-tale XY cells which raises the prospect of intergenerational microchimeric transfer [24]. In the simpler case, a mother might harbour Y chromosomes deriving from a male fetus, which in a subsequent pregnancy could be passed on to a female conceptus; alternately, in the case of a conceptus whose mother had only female pregnancies, male microchimeric cells could be transmitted from the mother’s own mother [13, 23, 47], in what is known as grandmaternal microchimerism. Going further, ‘the fetal microchiome in a mother is made up of a pool of cells contributed by her pregnancies and her matrilineal pregnancies across several generations’ [45]. In place of heritable parental genes originating in the sex specific gametes of the male and female parent which supposedly alone determine the sex of the embryo and subsequent child, any body could code for non-inherited DNA expressed by the opposite sex. Genetic inheritance is in any case far from definitive. As Donna Haraway has put it: ‘Short of cloning. neither parent is continued in the child, who is a randomly reassembled genetic package projected into the next generation’ [17].

Microchimerism can also be traced to the brain [43]. While the place of gonadal and hormonal sex differences in cognitive neuroscience is well-established [9], there is a growing acceptance that the human blood/brain barrier is, in fact, permeable and that non-self cells cross it. This allows for the emplacement of microchimeric cells of non-maternal/fetal origin such as those arising from transplantation and non-irradiated blood transfusions. The presence of Y-coded DNA embedded in the adult female brain – in possibly as many as 63% of women [6] – shifts the problematic from the phenotype to something far more nebulous and even existential. Microchimeric cells have been found in many areas of the brain, including those crucial for cognition, memory, and emotion [6], such that it is not just the biology of the body that is affected but behaviour and sense of self as well. Given that all bodies are most likely to be a mix of male and female genetic material, albeit one where one sexual marker usually outweighs the other, one plausible explanation for at least some of the many biological intersex conditions [10, 22, 27] is a skewing of that ratio to the extent that a form of macrochimerism is in place. The incidence of transgenderism is somewhat different in that it is not simply biological but a psychological, socio-cultural and environmental response that sets the parameters of what is manifested and/or internalised. In terms of development, the origins of any cell may influence the expression of genes such that sexual differentiation between the brains of men and women cannot be taken for granted. The bidirectional circulation of microchimerism, initially via the placenta, troubles that binary distinction and raises the question of what it means to be male or female, masculine or feminine. Both anatomical/gonadal/hormonal development and acquired gender identity may be uncertain, and, moreover, the two putatively distinct processes may be discordant.

Some researchers already argue that the presence of Y-coded DNA in female brains and *vice-versa* should radically refashion how sex differences in neurobiology are understood [15], but in general the research emphasis focuses on delineating another binary: that of harms and benefits. Johnson et al [21] do consider the issue with regard to sports activities, though in common with the plethora of others investigating microchimerism in the brain, they are more concerned with the functional implications for health and disease. Hanley’s work on what he calls dual-gender microchimerism [15] as evidenced in intersex and transsexuality is an exception, while Joel [19] takes up the question of sex differentiation in the brain but names the underlying cellular structure as mosaic rather than microchimeric. In brief, mosaicism indicates that individuals may have a patchwork of genetically distinct cell lines – explained by internal mutation - that originated from a single zygote, whereas chimerism denotes the allopathic origin of at least one cell line. In practice, however, the distinction between the two forms of anomaly is uncertain: a diagnosis of mosaicism cannot exclude chimerism and actually may be chimerism. Joel’s assertion, then, that ‘most brains are composed of unique mosaics of features, some in the form more common in females and some in the form more common in males. Moreover, these mosaic brains would not fit into two distinct categories, namely, male and female brains, nor would they be aligned along a male–female brain continuum’ [19], equally describes the case with microchimerism.

Joel’s overall point is that rather than typical male and female brains, the similarities and differences are not sex-determined and that brains are, in her words, multimorphic rather than dimorphic. Her major concern, however, as previously outlined by Joel and Fausto-Sterling [20] is with subsequent function which as she points out is complexly context-dependent. That path takes her away from my own point that although the functional status of microchimerism remains uncertain, it raises the philosophical issue – perhaps more ontological than empirical – of the implications of porous boundaries between putative sex/gender identities. Current work is still sparse and speculative (as Joel’s mosaic brain hypothesis itself illustrates), but at very least the possibility of sex-specific maternal microchimerism in the developing infant brain and fetal microchimerism in the mother’s brain warrants deeper investigation.

Like all authoritative discourses, human biology tends to invest in strategies of representation that angle the empirical data to correspond with a preconceived configuration. The advent of postgenomics, however, opens up a new fluidity that signals the possibility of a thoroughgoing reconfiguration. I suggest that the rapidly growing evidence of microchimerism does not fit the oppositional self/non-self paradigm – including that of biological sex – and throws into question the normative context in which the inviolability of clear boundaries between corporeal categories is taken as given. There is now wide acceptance that microchimerism is the rule rather than the exception, a finding explicitly endorsed by Dierselhuis and Goulmy [7], and more cautiously by others, including Lee Nelson [32], perhaps the most influential researcher in the field. Contrary to the expectation of genetic essentialism, microchimeric research reveals the self as a porous, unbounded entity, in effect an ecosystem of variable and unfixed influences. The binaries of self and other, male and female, sex and gender, normal and abnormal, parent and child are all disturbed. In the case of reproduction, the birth parent – and I include trans men here - shares non-inherited genes with both the children and the grandparent, as well as traces of siblings or previous pregnancies that have never come to term. The very existence of microchimerism and the probability that it is ubiquitous fundamentally contests any notion of singularity and speaks instead to a shared identity across normative categories. It is here that the biological sciences can come together with philosophy and sociocultural studies to overturn unwarranted binaries and fully advance an understanding of the chimerical nature of human co-existence.

## Data Availability

No datasets were generated or analysed during the current study.

## Footnotes

1 More radical terminology now refers only to *differences* in sexual development.

2 Tetragametic chimerism occurs when two zygotes fuse and develop into a single body with two distinct sets of DNA.

3 The allopathic model of biomedicine posits that all corporeal cells encompass the Human Leukocyte Antigen that marks them as unique self-cells. When a mature immune system encounters cells without similarly precise combinations – as in a fetus or transplant – they are identified as not-self and subjected to an immune response that treats the unrecognised material as pathogenic.

4 See Martin [28] for her revealing interviews with Bianchi about the difficulties she faced in getting her research accepted.
